# Problematic Internet Use among Adolescents 18 Months after the Onset of the COVID-19 Pandemic

**DOI:** 10.3390/children9111724

**Published:** 2022-11-10

**Authors:** Frank W. Paulus, Jens Joas, Ida Gerstner, Anna Kühn, Markus Wenning, Thomas Gehrke, Holger Burckhart, Ulf Richter, Alexandra Nonnenmacher, Michael Zemlin, Thomas Lücke, Folke Brinkmann, Tobias Rothoeft, Thorsten Lehr, Eva Möhler

**Affiliations:** 1 Department of Child and Adolescent Psychiatry, Saarland University Hospital, 66421 Homburg, Germany; 2 Department of Clinical Pharmacy, Saarland University, 66123 Saarbrücken, Germany; 3 Medical Association, Westfalen-Lippe, 48151 Münster, Germany; 4 Vaccination Center, 57072 Siegen, Germany; 5 School of Education and Psychology, Siegen University, 57072 Siegen, Germany; 6 Department of General Pediatrics and Neonatology, Saarland University Hospital, 66421 Homburg, Germany; 7 Department of Pediatrics, Ruhr University, 44791 Bochum, Germany

**Keywords:** problematic internet use, internet addiction, COVID-19, pandemic, emotional dysregulation, adolescent

## Abstract

Studies in recent years and especially since the beginning of the COVID-19 pandemic have shown a significant increase in the problematic use of computer games and social media. Adolescents having difficulties in regulating their unpleasant emotions are especially prone to Problematic Internet Use (PIU), which is why emotion dysregulation has been considered a risk factor for PIU. The aim of the present study was to assess problematic internet use (PIU) in adolescents after the third wave (nearly 1.5 years after the onset in Europe) of the COVID-19 pandemic. In the German region of Siegen-Wittgenstein, all students 12 years and older from secondary-level schools, vocational schools and universities were offered a prioritized vaccination in August 2021 with an approved vaccine against COVID-19. In this context, the participants filled out the Short Compulsive Internet Use Scale (SCIUS) and two additional items to capture a possible change in digital media usage time and regulation of negative affect due to the COVID-19 pandemic. A multiple regression analysis was performed to identify predictors of PIU. The original sample consisted of 1477 participants, and after excluding invalid cases the final sample size amounted to 1268 adolescents aged 12–17 (x = 14.37 years, SD = 1.64). The average prevalence of PIU was 43.69%. Gender, age, digital media usage time and the intensity of negative emotions during the COVID-19 pandemic were all found to be significant predictors of PIU: female gender, increasing age, longer digital media usage time and higher intensity of negative emotions during the COVID-19 pandemic were associated with higher SCIUS total scores. This study found a very high prevalence of PIU among 12- to 17-year-olds for the period after the third wave of the COVID-19 pandemic, which has increased significantly compared to pre-pandemic prevalence rates. PIU is emerging as a serious problem among young people in the pandemic. Besides gender and age, pandemic-associated time of digital media use and emotion regulation have an impact on PIU, which provides starting points for preventive interventions.

## 1. Introduction

The access to information and communication technologies (ICT) has constantly risen among adolescents over the past years. Due to the deprivation of normal activities, social isolation, lockdown and home-schooling during the coronavirus pandemic (COVID-19), the frequency of ICT use and its ramifications are of vital importance, now more than ever [1,2,3]. Specific, pronounced internet-related problems concomitant with this development seem to have appeared and additionally increased in recent years: gaming disorder, social network disorder, excessive internet shopping, internet pornography and online sex addiction, cyber-grooming and cyber-mobbing. Nowadays, there are no formal diagnostic criteria available for specific internet-related problems neither in the International Classification of Diseases and Related Health Problems (ICD-10), nor in the Diagnostic and Statistical Manual of Mental Disorders (DSM-5) (with a single exception: gaming disorder in the ICD-11). Nevertheless, different taxonomical terms exist in the literature describing the phenomenon concerned in general terms, including smartphone addiction, net compulsions, internet dependency, internet addiction (IA), compulsive internet use (CIU) or problematic internet use (PIU) [4,5,6,7,8].

Problematic internet use (PIU) as a generalized concept [9] encompasses various specific internet-related activities, as outlined above. It is described by criteria including: (1) experiencing unpleasant emotions when internet use is impossible, (2) continuing internet use despite the intention or desire to stop the use, (3) using the internet to ameliorate negative emotions, (4) internet use dominating one’s thoughts and behaviors and (5) internet use resulting in inter- or intra-personal conflicts [10]. PIU includes not only pronounced disorders, but many forms of precursors, both milder forms of disturbance and risky use as precursors to severe disturbance as well as continued harmful or abusive use that is associated with negative consequences. In addition to PIU, IA is a comparably cross-domain and superordinate construct [11,12] for different forms of (non-substance-related) behavioral addictions that do not focus on specific areas such as gaming or social communication.

Currently, the only disorder characterized in the DSM-5 and the ICD-11 strongly related to and a part of PIU/IA is (computer/video/tablet/smartphone) IGD/GD: internet gaming disorder (IGD) is a diagnosis in the Section III (conditions for further study) of the DSM-5 [13], whereas gaming disorder (GD) is classified as a clinical diagnosis in the ICD-11 [14]. Other forms of PIU, for instance the problematic use of SM (e.g., Facebook, Instagram, Reddit, WhatsApp, YouTube, Tik Tok, Twitter), have not yet been included in the current version of the DSM or ICD. However, the DSM-5 criteria of IGD could be appropriate in clinically assessing other specific forms of PIU [15] such as social network use, internet pornography use or online shopping.

Screening tests for the general identification of PIU are the Compulsive Internet Use Scale (CIUS) [16] or the Internet Addiction Test (IAT) [4]. Regarding the prevalence, in a German version of the CIUS, PIU was found in 13.9% of adolescents [17]. Applying the IAT, internet use among adolescents between 13 and 17 in Lebanon showed that 40% suffered from a moderate level of internet addiction, whereas 3.6% showed a severe dependence on the internet [18]. In line with these findings, Huang and Shen state that adolescents are a high-risk group for developing PIU/IA [19].

PIU increases significantly with a daily internet usage time of over 2 h and results in less social support and more difficulties in the identification and verbal expression of feelings. It is also associated with decreases in a range of neuropsychological domains such as attentional inhibition, motor inhibition, or working memory [20,21,22,23]. In addition, comorbid disorders with PIU in adolescents are frequent, with attention-deficit hyperactivity disorder, social phobia, insomnia and major depressive disorder being most strongly represented [24,25,26]. Furthermore, it is a known fact that deficits in emotion regulation can influence disorders, including different substance use disorders and behavioral addictions [27,28,29]. Therefore, maladaptive coping mechanisms and emotion dysregulation can increase the risk of PIU [30,31,32].

Several studies found cyberbullying to be related to PIU [33] as well as a link between fear of missing out (FoMO) and PIU [9]. FoMO, the urge to check one’s phone for new messages or updates and the concern of being left out and of missing out, appears to be one additional reason why adolescents are engaged in problematic internet use [34]. Furthermore, PIU (smartphone use) might cause no mobile phone phobia (NMP), a health problem that has a negative effect on mental, physical and social health [35,36].

Regarding previous literature, gender differences in PIU are still unclear and culturally determined [37]. No differences in PIU between boys and girls were found with the CIUS-9 [38].

The relation between PIU, generalized social beliefs and emotional problems plays an important role in the treatment for PIU (cognition-based intervention strategies for reducing PIU), especially since the COVID-19 outbreak [39]. Children who experienced quarantine in other pandemics report higher depressive and stress symptoms. Stress can have negative consequences on emotional and cognitive functions, such as decreases in self-esteem over time [40,41]. Therefore, internet consumption increases when people feel lonely, depressed, anxious, or desire emotional support [42]. Family involvement can thereby serve as a protective factor against PIU [43].

Especially during the COVID-19 pandemic, the risk of developing PIU in youth has increased [44,45]. In Taiwan, for example, the prevalence rate among adolescents was 17.4% before the pandemic, while it increased to 24.4% during the pandemic [46,47].

The aim of the present study was to assess the prevalence of PIU in a representative cohort of adolescents aged 12–17 after one and a half years of the COVID-19 pandemic. Additionally, we examined the influence of age and gender on the PIU prevalence. Furthermore, we investigated the influence of pandemic-related changes in digital media usage time and emotion regulation strategies (restlessness, irritability, anger, anxiety, or sadness) on PIU when digital media could not be used.

## 2. Method

### 2.1. Study Design

The present study is part of a pilot project conducted in the Siegen-Wittgenstein region from July to September 2021 (the actual data collection took place in the period from 30 July to 30 September 2021). The study was conducted by the University of Siegen with the vaccination center Siegen in collaboration with other investigators: University of Saarland (Department of Clinical Pharmacy), University Children’s Hospital Bochum, Saarland University Hospital Clinic for General Pediatrics and Neonatology and Saarland University Hospital Clinic for Child and Adolescent Psychiatry, Psychosomatics and Psychotherapy.

Adolescents and their caregivers were offered a prioritized SARS-CoV-2 vaccination (BNT162b2 by Biotech/Pfizer). This was announced in local newspapers, radios and on the homepage of the University of Siegen. All interested adolescents and families could participate.

In this context, supplementary data were collected in a survey, and participation in the survey was independent of receiving the vaccination. The survey included questions on sociodemographic data, history of COVID-19 infection and the vaccination status of the adolescent. Further, participants were invited to complete questions on PIU, on changes in their temporal usage behavior and on regulation of negative feelings during the pandemic. The results of further questions on mental health and health-related quality of life have already been published [48].

All participants and their caregivers were informed and provided written consent prior to their participation in the survey. Compliance with the Declaration of Helsinki and approval of the Ethics Committee of the Medical Association of Westphalia-Lippe and the Westphalian Wilhelms University were obtained (file number: 021-372-f-S).

### 2.2. Participants

**Figure 1:**
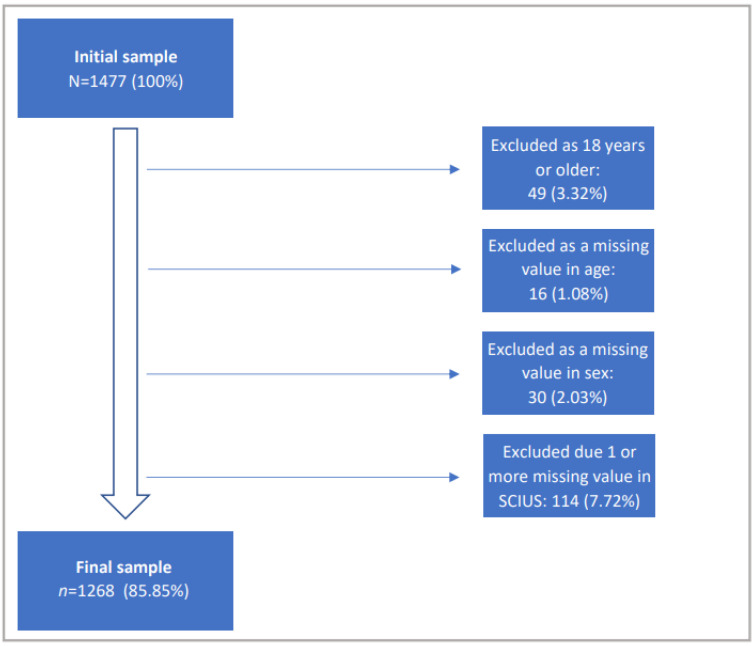
Flow of participants.

The present sample was an ad-hoc sample. Inclusion criteria for this study were as follows: (a) minimum age of 12 years, (b) attending a secondary school or vocational school in the district of Siegen or a matriculation at the University of Siegen, (c) COVID-19 vaccination and (d) voluntary participation in the survey. Exclusion criteria were based on contraindications to the vaccine, such as an age under 12 years because of the lack of an approved vaccine for this age group as well as a known hypersensitivity to any vaccine ingredient (for all contraindications see the RKI information leaflet on vaccination against COVID-19 in the version of 29 November 2021). From an initial 1477 participants, 49 (3.3%) were excluded because of their age of 18 years and older, 16 (1.1%) showed a missing value in age, 30 (2.0%) had a missing value in gender and 114 (7.7%) were excluded due to 1 or more missing values in SCIUS (see chapter measures). Thus, the final sample size was 1268 participants, 85.9% of the initial sample. Participant flow can also be taken from Figure 1. The recruitment and education about the study took place in the vaccination center. The questionnaires were voluntarily filled out online on a tablet provided by the study leaders or via a QR-Code or internet address on the participants’ own mobile terminal in the medically determined waiting period after the vaccination.

### 2.3. Measures

The Compulsive Internet Use Scale (CIUS) is a validated self-report questionnaire measuring the severity of internet addiction on a continuum of 0 to 56 points. The scale consists of 14 items and the response options are presented in a 5-point Likert scale. The CIUS shows a good factorial stability, a high internal consistency and therefore good reliability as well as good validity [16,49,50,51,52]. Furthermore, there are some reliable short forms of the CIUS such as the short French version of the CIUS with 9 items [38] and valid 5-, 7- and 9-item versions of the Lithuanian CIUS [53].

The SCIUS (Short Compulsive Internet Use Scale) used in this study is a short form of the CIUS. It consists of 5 of the original 14 items (see Table 1) rated with a 5-point Likert scale with the response options:”0 = never, 1 = seldom, 2 = sometimes, 3 = frequent, 4 = very frequent”. The scale assesses PIU as a short screening procedure. Regarding the test quality, the SCIUS has an acceptable reliability with a Cronbach’s Alpha of 0.77 for internal consistency. In addition, there is no significant deviation from the original CIUS regarding specificity and sensitivity [54]. As a cut-off for PIU, the value 7 (sensitivity = 0.95, specificity = 0.86 for females and 0.87 for males) or for a higher specificity (0.96) the value 9 (sensitivity = 0.76 for females and 0.78 for males) could be used according to the manual [54]. In our analysis, we used only the cut-off value 9 for a higher specificity.

Furthermore, to capture the change due to the COVID-19 pandemic, 2 more items were introduced. The first item captures digital media usage time: “To what extent does the COVID-19 pandemic change the time you use digital media (smartphone, smartwatch, tablet, laptop, stationary/portable game console)?” The response options were in the form of a five-point Likert scale: Since then I have been using digital media...”much less/much shorter”, “somewhat less”, “same amount/unchanged”, “more often/longer”, “much more often/longer”. The second item asks about the intensity of negative feelings due to the COVID-19 pandemic: “To what extent does the COVID-19 pandemic change negative feelings (e.g., restlessness, irritability, anger, anxiety, or sadness) when you do not have the opportunity to use digital media?” Again, the response options were given with a five-point Likert scale: I have been reacting in the intensity of my negative feelings since then...“much less strongly/severely”, “less strongly/severely”, “equally/unchanged”, “more strongly/more severely”, “much more strongly/severely”.

## 3. Results

### 3.1. Sample

The final sample size consisted of 1268 participants, 85.9% of the initial sample. The age range was from 12 to 17 years (M = 14.37, SD = 1.64), with 650 female (51.3%), 608 male (48%) and 10 X-gender (0.8%) participants. Further, 58 adolescents (4.6%) of the sample and 210 parents (16.6%) were born outside of Germany. In addition, 77 (6.1%) respondents mentioned one or more chronic diseases or disabilities, with by far the most frequent disease being bronchial asthma (29.4%), followed by ADHD (8.2%) and diabetes (7.1%). Epilepsy (5.9%) and allergy (4.7%) were in fourth and fifth place, respectively. For a complete depiction of all sample characteristics, see Table 2.

### 3.2. Exploratory Factor Analysis

**Figure 2:**
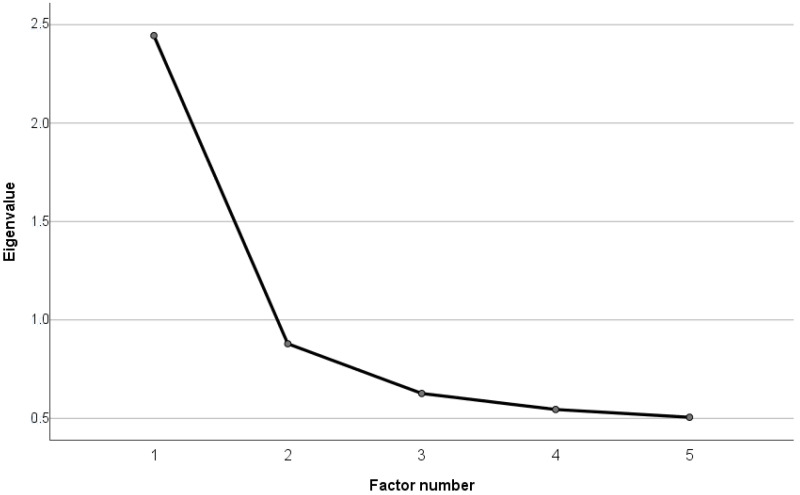
Results of scree plot of explanatory factor analysis of SCIUS.

The structure of the short form of the Compulsive Internet Use Scale (SCIUS) to assess problematic internet use (PIU) (5 items) was examined using an exploratory factor analysis on the sample of 12- to 17-year-old adolescents. Both Bartlett’s test ($\chi^{2}$(10) = 1250.15, *p* ≤ 0.001) and the Kaiser–Meyer–Olkin Measure of Sampling Adequacy (KMO = 0.78 (middling according to Kaiser and Rice [55])) indicated that the variables were suitable for a factor analysis. Thus, a principal component analysis with varimax rotation was performed. The result was one factor with eigenvalue > 1.0. The graphical representation in the form of a scree plot (see Figure 2) also suggested a one-factor solution, which explains 48.89% of the variance. The factor appears interpretable according to the criteria of Guadagnoli and Velicer [56], since 4 items load >0.60 on the factor. In terms of content, naming this factor “PIU” is conceivable, as it elicits topics such as dysfunctional internet use or negative consequences of internet use. Further information can be found in Table 1.

### 3.3. Item Analysis and Reliability

Following Ebel and Frisbie [57], items 1–4 of the SCIUS showed a very good discrimination power and item 5 a reasonably good discrimination power. The item difficulty was in the acceptable range for all items. Cronbach’s α is 0.73 and thus acceptable according to George and Mallery [58]. Detailed item statistics are shown in Table 1.

### 3.4. Descriptive Prevalence Statistics: SCIUS

In total, 43.69% of the participants showed problematic internet use (PIU). It should be emphasized that more girls than boys were above the cut-off value (female = 49.38%, male = 37.50%). Furthermore, there was a small difference between the group of early adolescents (12–14 years) (42.06%) and the group of late adolescents (15–17 years) (45.58%). In terms of digital media usage time, PIU was found in 53.27% of those who had a higher digital media usage time in the pandemic, but only in 29.81% of those who used digital media less in the pandemic than they did before.

Moreover, 66.67% of the participants who reported a higher intensity of negative emotions during the COVID-19 pandemic, if there was no possibility to use digital media, reached the criteria for PIU, but only 39.38% of those reporting a lower intensity of negative emotions during the COVID-19 pandemic did. See Table 3 for further information.

### 3.5. Descriptive Statistics: COVID-19 Items Usage Time and Emotion Regulation

Out of 1268 participants, 104 (8.21%) indicated that their digital media usage time was much less/much shorter during the COVID-19 pandemic, whereas 734 (57.89%) indicated having a longer or much longer digital media usage time during the COVID-19 pandemic. Regarding the intensity of negative emotions during the COVID-19 pandemic, 160 (12.61%) mentioned that they have been reacting in the intensity of their negative feelings less strongly or much less strongly, and 303 (23.9%) more strongly or much more strongly during the COVID-19 pandemic. See Table 4 for further data.

**Table 3:** Descriptive statistics of SCIUS.

	*N*	Items	M	SD	Minimum	Maximum	*n ≥* Cut-Off	*% ≥* Cut-Off
Total	1268	5	8.07	4.03	0	20	554	43.69
Male	608	5	7.57	3.88	0	20	228	37.50
Female	650	5	8.50	4.10	0	20	321	49.38
X-Gender	10	5	11.00	3.86	7	19	5	50.00
Early adolescents (12–14 years)	680	5	7.88	4.13	0	20	286	42.06
Late adolescents (15–17 years)	588	5	8.29	3.90	0	19	268	45.58
Shorter digital media usage time	104	5	6.53	3.98	0	19	31	29.81
Longer digital media usage time	734	5	8.99	3.94	0	20	391	53.27
Lower intensity of negative emotions during the COVID-19 pandemic	160	5	7.28	3.77	0	19	63	39.38
Higher intensity of negative emotions during the COVID-19 pandemic	303	5	10.40	4.03	0	20	202	66.67

Note: “Shorter digital media usage time” = sum of the values of the categories “much less/much shorter” and “somewhat less”. “Longer digital media usage time” = sum of the values of the categories “more often/longer” and “much more often/longer”. “Lower intensity of negative emotions during the COVID-19 pandemic” = sum of the values of the categories “much less strongly/severely” and “less strongly/severely”. “Higher intensity of negative emotions during the COVID-19 pandemic” = sum of the values of the categories “more strongly/more severely” and “much more strongly/severely”.

### 3.6. Influence of Gender, Age, Usage Time and Emotion Regulation on SCIUS

Gender was limited to male and female, as the group of X-genders was too small, with 10 cases. Girls (M = 8.50, SD = 4.10, *n* = 650) scored higher SCIUS total scores than boys (M = 7.57, SD = 3.88, *n* = 608). A Mann–Whitney U-test indicated that this difference was statistically significant (*U*(*n*_girls_ = 650, *n*_boys_ = 608) = 171,952.50, *z* = −3.99, *p* ≤ 0.001). The effect size according to Cohen [59] was Pearson *r* = 0.11, and it corresponds to a small effect.

Late adolescents, defined as age 15–17 years (M = 8.29, SD = 3.90, *n* = 588), showed higher SCIUS total scores than early adolescents, defined as age 12–14 years (M = 7.88, SD = 4.13, *n* = 680). This difference was also statistically significant (*U*(*n*_early adolescents_ = 680, *n*_late adolescents_ = 588) = 186,472.00, *z* = −2.07, *p* = 0.04). The effect size here was Pearson *r* = 0.06 (small or no effect according to Cohen [59]).

Adolescents with a longer digital media usage time (answered “more often/longer” or “much more often/longer”) (M = 8.99, SD = 3.94, *n* = 734) showed higher SCIUS total scores than adolescents with a shorter digital media usage time (answered “somewhat less” or “much less/much shorter”) (M = 6.53, SD = 3.98, *n* = 104) (see Table 3). This distinction was significant, as determined by a Mann–Whitney U-test (*U*(*n*_longer digital media usage time_ = 734, *n*_shorter digital media usage time_ = 104) = 24,962.00, *z* = −5.73, *p* ≤ 0.001). The effect size was Pearson *r* = 0.20. Following Cohen [59], this is a small effect.

Adolescents with a higher intensity of negative emotions during the COVID-19 pandemic (answered “more strongly/more severely” or “much more strongly/severely”) (M = 10.40, SD = 4.03, *n* = 303) scored higher SCIUS total scores than adolescents with a lower intensity of negative emotions during the COVID-19 pandemic (answered “less strongly/severely” or “much less strongly/severely”) (M = 7.28, SD = 3.77, *n* = 160) (see Table 3). A Mann–Whitney U-test indicated that this difference was statistically significant (*U*(*n*_higher intensity of negative emotions_ = 303, *n*_lower intensity of negative emotions_ = 160) = 13,690.50, *z* = −7.72, *p* ≤ 0.001). In this case, the effect size is Pearson *r* = 0.36, which, according to Cohen [59], is equivalent to a moderate effect.

### 3.7. Regression Analysis

A multiple regression analysis was used to predict the SCIUS total score from gender (limited to male and female, as the group of X-genders was too small, with 10 cases), age, digital media usage time and intensity of negative emotions during the COVID-19 pandemic. The model explained a statistically significant amount of variance in the SCIUS total score, *F*(4, 1111) = 53.16, *p* ≤ 0.001, *R²* = 0.16, *R²*_adjusted_ = 0.16. All were significant predictors: gender (*ß* = −0.09, *t* = −3.40, *p* = 0.001), age (*ß* = 0.09, *t* = 3.18, *p* = 0.002), digital media usage time (*ß* = 0.28, *t* = 9.95, *p* ≤ 0.001), intensity of negative emotions during the COVID-19 pandemic (*ß* = 0.20, *t* = 7.05, *p* ≤ 0.001). Therefore, the final predictive model was: SCIUS total score = 0.93 − 0.76 (gender) + 0.22 (age) + 1.22 (digital media usage time) + 1.00 (intensity of negative emotions during the COVID-19 pandemic). Female gender, increasing age, longer digital media usage time and higher intensity of negative emotions during the COVID-19 pandemic were associated with higher SCIUS total scores. The *R²* for the overall model indicates a moderate goodness of fit according to Cohen [59], *f²* = 0.19 (medium effect). See Table 5 for further multiple regression results.

## 4. Discussion

The present study aimed to assess problematic internet use (PIU) among adolescents after the third wave of the COVID-19 pandemic. In an online study, the SCIUS was used to measure PIU among adolescents aged 12 to 17. In addition, changes in digital media usage time and intensity of negative emotions during COVID-19 were assessed. Multiple regression revealed that gender, age, digital media usage time and intensity of negative emotions during the COVID-19 pandemic were all significant predictors of PIU. Thus, female gender, increasing age, longer digital media usage time and higher intensity of negative emotions during the COVID-19 pandemic were associated with a higher PIU.

In summary, the research questions of this study were to determine the prevalence of PIU and to investigate the influence of gender and age on the prevalence of PIU. Additionally, the influence of pandemic-related changes in digital media usage time and emotion regulation strategies (restlessness, irritability, anger, anxiety, or sadness) on PIU when digital media could not be used were examined.

### 4.1. Prevalence of PIU

A growing prevalence of excessive internet use has been described in most industrialized countries (especially Asian, European and North American countries).

One of the main findings of our study is that one and a half years after the start of the COVID-19 pandemic, a prevalence of PIU of 43.69% was found in adolescents aged 12–17 years.

This value is very high compared to other studies, conducted before the pandemic, with a prevalence range of 10–24% [60,61,62,63] (data collection period 2017–2018). Overall, prevalence rates in adolescents start from 1.5% in Greece, 10.7% in South Korea to 11.6% in Latin America [64,65,66]. In Hong Kong, prevalence rates among secondary students are estimated to even reach up to 20% [67]. Comparably high values were found in a Spanish study: 24% of adolescents between 14 and 18 used the internet in a problematic way, with the intensity being highest among those between 16 and 17 years [68]. Chandrima et al. also reported that 24.0% of adolescents from Bangladesh were problematic internet users and 2.6% had severe PIU [23].

**Figure 3:**
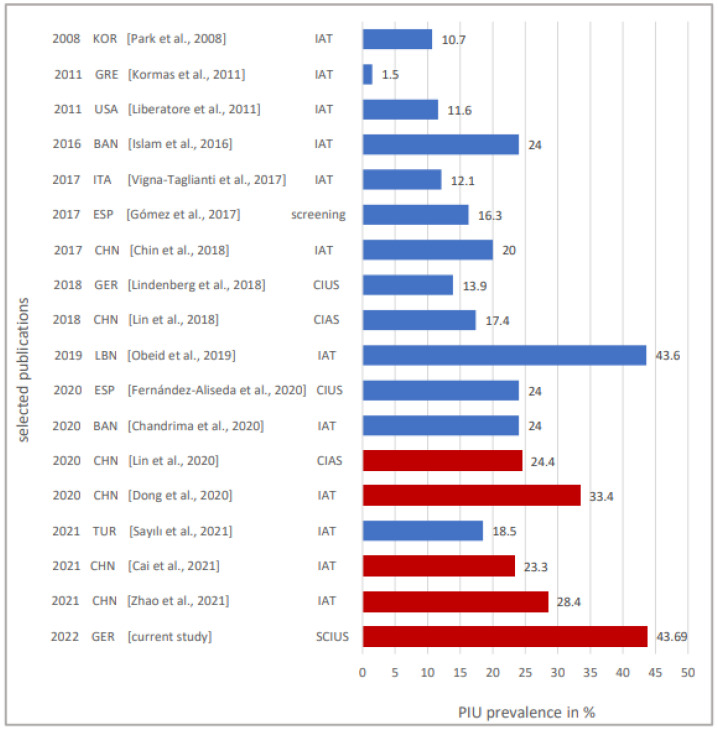
Selected PIU prevalence rates, depending on the year of publication, country the data were collected in and measurement instrument used. Column 1: year of publication, column 2: country (KOR = South Korea, GRE = Greece, USA = United States of America, BAN = Bangladesh, ITA = Italy, ESP = Spain, CHN = China, LBN = Lebanon, TUR = Turkey, GER = Germany), column 3: reference and column 4: measurement instrument used. Blue: data from before the pandemic were used, red: data from during the pandemic were used. Refs in figure are [17,18,23,46,47,60,61,62,63,64,65,66,67,68,69,70,77].

In summary, data from various studies conducted in different countries prior to the pandemic show prevalence rates of about 25% of PIU. One interpretation of the prevalence of PIU of 43.69% in adolescents aged 12–17 years after the third wave of the COVID-19 pandemic is that the data collections of the other studies almost all occurred before the COVID-19 pandemic. In contrast, the data of the present study were collected after the third wave of the pandemic, which has been in progress for one and a half years. Thus, the context of the pandemic (lockdown, school closures, reduction in real-world social contact with peers, increase in time spent using the internet) could have led to a significant increase in the prevalence of PIU, as shown by several other studies conducted during the pandemic with prevalence rates between 24% and 28% [47,69,70]. Oka et al. reported, in their study on internet gaming disorder and problematic internet use before and during COVID-19, that during the pandemic, the prevalence of IGD increased 1.6-fold and the prevalence of PIU increased 1.5-fold compared to before the pandemic [71]. An overview of a selection of previous PIU prevalence rates with the distinction “before” vs. “during” the pandemic, compared to the prevalence found in our study, can be found in Figure 3.

A major problem in estimating the prevalence of PIU is the lack of consensus on criteria and definitions, cut-off values and a unified terminology. It is also conceivable that the use of different measurement instruments to measure PIU (CIUS, SCIUS, IAT) plays a role. Regardless, the present study found an enormously high prevalence of 43% of PIU in a representative sample of adolescents one and a half years after the start of the pandemic.

A complementary explanation for the increase in the prevalence of PIU (at least for the increase in Germany) could be due to the policy of internet use in Germany.

On the one hand, the digitalization campaign currently being conducted by the German government creates a framework for a significant increase in the use of digital screen media. The “DigitalPakt Schule” (Digital Pact for Schools) aims to promote digitalization in German schools [72]. This brings many advantages, but at the same time also carries the risk of an increase in the prevalence of PIU, as the use of the internet is becoming more and more self-evident and extensive for children and young people in many areas of their everyday lives. The implementation strategies for shaping the digital transformation in Germany include five fields of action: digital competence, infrastructure and equipment, innovation and digital transformation, society in the digital transformation and modern state [73]. All these fields of action promote the use of digital media and at the same make digital media and the internet more accessible. This indicates the need for clear guidelines on the use of the internet and digital media, such as those issued by the Federal Centre for Health Education (BZgA) [74].

Additionally, the legislation of the Youth Protection Act (Second Act Amending the Youth Protection Act, 2021 [75]) has been assessed by pediatric professional associations to be in need of improvement [76]. The developmentally impairing consequences of excessive use of digital screen media (in terms of duration of use, device use and content) are insufficiently considered in the law. For example, it is urgently recommended to correct the current age rating of digital applications “from 0 years” to “from 3 years”. Scientific process support through developmental neurological and psychological research is lacking.

### 4.2. Gender and PIU

Gender predicted PIU. More girls than boys were above the cut-off for PIU (female = 49.38%, male = 37.50%). Girls scored higher on PIU than boys.

On the one hand, findings consistent with the results of this study, with females showing significantly higher PIU than males, were found by Laconi et al. and Mihara et al. [78,79]. In addition, differences were also found between males and females in terms of school- or work-related internet use, with female participants showing higher usage in this area [80].

On the other hand, many studies [64,81] show contrary findings, with males having significantly higher PIU than females and comparably higher prevalence rates for boys regarding IA [82,83,84,85,86,87] as well as a higher propensity for IA [88]. Especially male adolescents with low life satisfaction and low academic performance are more at risk for PIU [49].

However, there are also studies that found no correlation between gender and the prevalence of PIU for either internet use disorder [17], IAT [89,90,91] or CIUS [92].

Given these ambiguous findings, it can be speculated that the female dominance in PIU scores found here may be due to the significantly increased social media use during the pandemic, as several previous studies note a significant preference for computer games among boys and a preference for SM use among girls [93,94,95,96,97,98]. Nevertheless, further investigations of moderating effects regarding gender differences in PIU are needed.

### 4.3. Age and PIU

There was a small difference in the PIU prevalence between the group of early adolescents (12–14 years old) with 42.06% and the group of late adolescents (15–17 years old) with 45.58%. Age significantly predicted PIU. Late adolescents, defined as age 15–17 years, showed higher PIU than early adolescents (12–14 years).

In contrast, the study by Schimmenti et al. [99] did not find age to be a predictor of PIU. To summarize the findings to date on this topic, there have been inconsistencies across the literature, as some studies claim to have found an effect of age on PIU [47,82] and others that there is no such effect [100]. Another interesting aspect is the different possible understandings of PIU. Perhaps a meta-analysis summarizing and comparing the findings and aligning them with their exact diagnostic criteria could shed some light on the different findings on age.

In order to precisely pinpoint which age group is most prone to PIU, further research should focus on a large sample across multiple age groups. This could be helpful in determining at what age preventive measures should be taken and when they are most effective. In general, the finding of increased PIU among older youths compared to younger ones remains plausible based on the overall higher time use of the internet with increasing age, as well as a better availability of media devices and less regimentation by parents.

### 4.4. Digital Media Usage Time during COVID-19 Pandemic

PIU was found in 53.27% of those who had a higher digital media usage time during the pandemic, but only in 29.81% of those who used digital media less during the pandemic than before. Digital media usage time serves as a predictor of PIU. Adolescents with a longer digital media usage time during the pandemic scored higher on PIU than adolescents with a shorter digital media usage time during the pandemic.

This finding replicates prior work by Schimmenti et al. [99], who found time spent online being a predictor of PIU, too. Time of use and PIU are significantly related, but this cross-sectional study cannot clarify what is the cause and what is the effect. Nevertheless, this finding may help in both prevention and treatment of PIU. Furthermore, Lai et al. [82] confirmed that the amount of time spent online is a risk factor for IA, going so far as to say that one additional hour spent online already increases addiction or problematic behavior. Independent of the pandemic and specific to gaming, Gentile et al. showed in a longitudinal design that more time spent gaming is a significant predictor of a subsequent gaming disorder (GD) [101]. In contrast, Yildiz Durak found no significant correlation between duration of social media usage and problematic social media usage [102]. However, it should be noted that this study refers specifically to problematic social media usage and not to PIU in general, which could explain the different results.

In the face of the pandemic, these results concerning digital media usage time become relevant due to the increasing amount of time children and adolescents spend online, with significantly higher rates of dependency following [103]. Related to the higher digital media usage time during the COVID-19 pandemic found in the study presented here, Eales et al. found a significant increase in screen media use and problematic media use among children in the United States during the COVID-19 pandemic [104]. Drouin et al. noted an increase of SM usage during COVID-19 [105]. The increase in the time adolescents spend using games and SM increases the risk of problematic patterns of use [106]. It would be of great interest to undertake further research on prevention measures, e.g., time limitation.

### 4.5. Intensity of Negative Emotions during the COVID-19 Pandemic

As a transdiagnostic construct, emotional dysregulation (ED) encompasses the inability to regulate the intensity and quality of emotions. Regulation of one’s own emotions is important in eliciting adequate emotional responses, dealing with excitability, mood instability and emotional over-reactivity, as well as returning to an emotional baseline [31]. A total of 66.67% of the participants who reported a higher intensity of negative emotions during the COVID-19 pandemic (if there was no possibility to use digital media) reached the criteria for PIU, but only 39.38% of those reporting a lower intensity of negative emotions during the COVID-19 pandemic did. This increased intensity of negative emotions during the COVID-19 pandemic predicted PIU: adolescents with a higher intensity of negative emotions during the COVID-19 pandemic scored higher on PIU than adolescents with a lower intensity of negative emotions during the COVID-19 pandemic. Therefore, one might conclude that the internet is used to regulate negative emotions and/or that PIU promotes ED, such as difficulties in recognizing emotions.

Morahan-Martin et al. found that internet utilization increases when people are feeling lonely [42]. Several studies found associations between PIU, anxiety, psychological distress and depression during COVID-19 [45,77,107]. Adolescents with higher levels of anxiety are more likely to increase their internet use [105]. Equally, Mamun et al. found an association between PIU and loneliness as well as psychological distress [108]. Social distancing and greater anxiety due to the COVID-19 pandemic led to an increased technology and social media use among children as well as parents [105]. Moreover, stronger emotional distress [109] and negative affectivity [99] were found to be predictors for PIU. Especially children with emotion dysregulation are at risk of problematic technology use, and in turn, PIU may lead to emotion dysregulation [31]. The correlation between emotion dysregulation and internet addiction has been identified several times across the literature [110]. Adolescents with PIU have more difficulties in identifying and describing emotions, understanding emotional reactions and controlling spontaneous behavior when negative emotional experiences occur [22].

This may result in a vicious cycle, making both the PIU and the emotion dysregulation more severe. Paulus et al. also prescribe different studies that all identify different aspects of emotion dysregulation as predictors of gaming disorder across different age groups [32]. They clearly outline the predictors of poor impulse control, and a lack of social skills as one of those predictors. Other studies suggest PIU being responsible for emotion dysregulation rather than vice versa [111]. They also specify that PIU only affects the more complex forms of emotion dysregulation such as pursuing life goals rather than impulse control. These findings about emotion dysregulation and PIU call for a closer investigation of these variables in the period of the current pandemic, as this is known to affect both variables independently [2,28].

The pandemic has led to significant stress and an increase in emotional disorders as well as an increase in PIU. PIU is closely related to emotion regulation strategies: those who reported having more negative emotions when they cannot use digital media during the pandemic also had significantly higher levels of PIU.

## 5. Limitations

A relevant issue is the range of possible measurement instruments to measure PIU. In this study, only a short version of the CIUS (SCIUS [54]), which consists of 5 of the original 14 items, was used. Whereas in other studies, for example, different CIUS versions or the IAT were applied [4]. The use of varying instruments to measure the same concept (PIU) could affect the results, which may be a possible explanation for the wide range of prevalence rates of PIU found across different studies. Second, this study only collected data from 12- to 17-year-old adolescents. However, as far as the relationship between age and PIU is concerned, there are still inconsistencies in research. To gain a better overview of which age groups are particularly affected by PIU, it would be necessary to conduct a study with a large sample across multiple age groups. Third, the data collected in this study are cross-sectional, hence the study design precludes conclusions about the extent to which the pandemic itself impacted PIU for the study sample. In addition, this study examined PIU in general. To gain a more in-depth insight into the relationship between gender and PIU, a more detailed survey on sub-areas of PIU (specific PIU) such as problematic gaming or problematic social media use would be useful. Furthermore, the SCIUS is a self-report questionnaire. Therefore, response biases such as wrong answers, socially desirable answers or under- and over-statements cannot be ruled out. As the questionnaires were also filled out by parents, exaggerations would also be possible here, since the behavior could be ubiquitous for the parents in everyday life and associated with negative feelings.

Moreover, although we searched thoroughly, the studies we found on PIU prevalence rates (pre-/post-COVID) do not claim to be exhaustive.

## 6. Outlook

Prevention on the three levels of preventive interventions: universal, selected and indicated prevention [112], should be reinforced to reduce PIU, including school-based preventive interventions. Regarding future prevention, limiting the time spent on internet activities could be a promising approach. Children prone to difficulties in dealing with negative emotions should be restricted in their internet use or monitored more closely. At the same time, more adequate coping and action alternatives should be offered that both act as an adaptive strategy for dealing with negative emotions as well as contribute to the experience of positive emotions [32].

Specific, pronounced internet-related problems or disorders such as gaming disorder or social network disorder, excessive internet shopping or internet pornography should be treated as well. These treatments could include focusing on a variety of non-screen time activities, as the lack of face-to-face activities offered leads to an increase in internet usage [103]. Measures can come from either the adolescents themselves, their parents, as well as their schools [113]. Adolescents can make sure to have a structured routine or to learn about emotional regulation strategies on their own. Parents could reduce general access to the internet, especially to younger children, or restrict certain websites to protect their children from harmful information. Schools that nowadays teach online could try to reduce screen time by assigning offline homework and by including mental health education in their templates.

The effects of the digitalization of society on child development have not yet been investigated.

## 7. Conclusions

The present study found a very high prevalence of 43.69% of PIU among 12- to 17-year-olds for the period after the third wave of the COVID-19 pandemic, which has increased significantly compared to pre-pandemic prevalence rates. Beside gender and age, pandemic-associated time of digital media use and emotion regulation were found to have an impact on PIU: the time criterion and emotional dysregulation could be assumed to be risk factors for the development of PIU.

What is needed is a consistent and competent education of parents, children and young people by medical, psychological and psychotherapeutic professionals about the health consequences of too-early and too-excessive media consumption. A reflective, critical, self-determined and meaningful use of electronic/digital media (devices) and the content accessible through those devices regarding media literacy (and not just media competence oriented towards feasibility) is the desirable and primary goal.

## Figures and Tables

**Table 1:** Item analysis statistics and results of explanatory factor analysis of SCIUS (*n* = 1268, items = 5). EV = Eigenvalue, VA = % of total variance explained, *h*^2^ = communality, * *p* < 0.05.

Item No.	Item	M	SD	Factor 1 EV = 2.44 VA = 48.89%	* h² *	Inter-Item Correlation	Item Difficulty	Discrimination Power	Cronbach’s α	Cronbach’s α, If Item Is Removed from the Scale
1	How often do you find it difficult to stop using the internet when you are online?	2.04	1.08	0.77	0.59	2: 0.40 * 3: 0.41 * 4: 0.46 * 5: 0.37 *	0.51	0.58	0.73	0.65
2	How often do other people (parents, friends) say you should use the internet less?	1.68	1.19	0.59	0.35	1: 0.40 * 3: 0.19 * 4: 0.37 * 5: 0.20 *	0.42	0.38		0.72
3	How often do you sleep too little because of the internet?	1.13	1.19	0.70	0.49	1: 0.41 * 2: 0.19 * 4: 0.42 * 5: 0.38 *	0.28	0.49		0.68
4	How often do you neglect your daily obligations because you prefer to go online?	1.21	1.05	0.76	0.58	1: 0.46 * 2: 0.37 * 3: 0.42 * 5: 0.37 *	0.30	0.57		0.65
5	How often do you go online when you are feeling down?	2.02	1.29	0.66	0.43	1: 0.37 * 2: 0.20 * 3: 0.39 * 4: 0.37 *	0.51	0.45		0.70

Data were analyzed using the IBM SPSS Statistics version 26. First, the characteristic values of the SCIUS were calculated for our sample. We chose the value 9 as the cut-off value for PIU in SCIUS, as it has a high specificity. For the analysis on PIU, *t*-tests were performed. If the requirements for a *t*-test for independent samples were not met, a Mann–Whitney U-test was carried out. Finally, a multiple regression analysis was conducted to predict PIU. A significance level of 0.05 was used for all statistical tests.

**Table 2:** Characteristics of the sample (*n* = 1268). MV = missing value.

Characteristic	*n*	%
**Child born outside Germany**		
MV	1	0.08
Child born outside Germany	58	4.57
**Parents born outside Germany**		
MV	2	0.16
Parent(s) born outside Germany	210	16.56
Both parents born outside Germany	145	11.44
Only mother born outside Germany	16	1.26
Only father born outside Germany	17	1.34
Only one parent born outside Germany, unspecified	15	1.18
No further information given	17	1.34
**Newborn gender**		
Male	608	47.95
Female	650	51.26
X-gender	10	0.79
**Chronic disease or disability**		
MV	3	0.24
None	1180	93.06
Respondents who mentioned one or more chronic diseases or disabilities	77	6.07

**Table 4:** Frequency data for the items digital media usage time and intensity of negative emotions during the COVID-19 pandemic.

Digital Media Usage Time	* n *	%	Intensity of Negative Emotions during COVID-19 Pandemic	* n *	%
Much less/much shorter	48	3.79	Much less strongly/severely	58	4.57
Somewhat less	56	4.42	less strongly/severely	102	8.04
Same amount/unchanged	387	30.52	Equally/unchanged	685	54.02
More often/longer	545	42.98	More strongly/more severely	259	20.43
Much more often/longer	189	14.91	Much more strongly/severely	44	3.47
MV	43	3.39	MV	120	9.46

**Table 5:** Multiple regression results using ICT temperament traits as predictors of SDQ.

Criterion	Predictor	B	SE B	β	T	*p*	Fit/R²	F	*p*
SCIUS Total Score							0.16	53.16	<0.001 *
	gender	−0.76	0.22	−0.09	−3.40	<0.01 *			
	age	0.22	0.07	0.09	3.18	<0.01 *			
	digital media usage time	1.22	0.12	0.28	9.95	<0.001 *			
	intensity of negative emotions during COVID-19 pandemic	1.00	0.14	0.20	3.18	0.01 *			

Note: *n* = 1115, smaller sample size resulting from missing values of the items digital media usage time and intensity of negative emotions during the COVID-19 pandemic. B represents unstandardized regression weights, SE B the standard error for B. Beta indicates the standard regression weights. * *p* < 0.05.

## Data Availability

The data that support the findings of this study are available from E. Moehler, but restrictions apply to the availability of these data, which were used under license for the current study and therefore are not publicly available. Data are, however, available from the authors upon reasonable request and with the permission of E. Moehler.
